# Increased serum levels of circulating exosomal microRNA-373 in receptor-negative breast cancer patients

**DOI:** 10.18632/oncotarget.2520

**Published:** 2014-10-11

**Authors:** Corinna Eichelser, Isabel Stückrath, Volkmar Müller, Karin Milde-Langosch, Harriet Wikman, Klaus Pantel, Heidi Schwarzenbach

**Affiliations:** ^1^ Department of Tumor Biology, University Medical Center Hamburg-Eppendorf, Germany; ^2^ Clinic of Gynecology, University Medical Center Hamburg-Eppendorf, Hamburg, Germany

**Keywords:** cell-free miRs, exosomal miRs, estrogen receptor, luminal breast cancer

## Abstract

In this study, we compared the blood serum levels of circulating cell-free and exosomal microRNAs, and their involvement in the molecular subtypes of breast cancer patients. Our analyses on cell-free miR-101, miR-372 and miR-373 were performed in preoperative blood serum of 168 patients with invasive breast cancer, 19 patients with benign breast diseases and 28 healthy women. MicroRNAs were additionally quantified in exosomes of 50 cancer patients and 12 healthy women from the same cohort. Relative concentrations were measured by quantitative TaqMan MicroRNA assays and correlated to clinicopathological risk factors. The concentrations of cell-free miR-101 (p=0.013) and miR-373 (p=0.024) were significantly different between patients with breast cancer and benign tumors. A prevalence of miR-101, miR-372 and miR-373 were found in exosomes. The levels of circulating exosomal (but not cell-free) miR-373 were higher in triple negative than luminal carcinomas (p=0.027). Also, estrogen-negative (p=0.021) and progesterone-negative (p=0.01) tumors displayed higher concentrations of exosomal miR-373 than patients with hormone-receptor positive tumors. Overexpression of miR-373 by transfection of MCF-7 cells showed downregulated protein expression of the estrogen receptor, and inhibition of apoptosis induced by camptothecin. Our data indicate that serum levels of exosomal miR-373 are linked to triple negative and more aggressive breast carcinomas.

## INTRODUCTION

Profiling by immunohistochemical assays using estrogen receptor (ER), progesterone receptor (PR), and human epidermal growth factor receptor 2 (HER2) classifies breast cancer into four phenotypically distinct subtypes: luminal A, luminal B, basal-like and HER2^+^ tumors. The quantification of the proliferation marker Ki-67 also supports this classification, which is a commonly accepted clinical adaption of the subtypes originally described by molecular analysis [1]. The expression pattern of luminal subtypes partially resembles that of the luminal epithelial layer of the mammary gland. The luminal A subtype is ER^+^ and/or PR^+^ and HER2^−^, with low Ki-67 levels and the luminal B subtype is ER^+^ and/or PR^+^ and either HER2^−^ with a high Ki-67 value or HER2^+^ with any Ki-67 value. Basal-like tumors display an expression pattern of basal myoepithelial markers and are mainly triple negative, thus ER^−^, PR^−^ and HER2^−^. They account for about 10–15% of all invasive breast cancers. The HER2^+^ subtype is ER^−^, PR^−^ and HER2^+^. The subtypes are associated with different clinical features and outcomes and predictive of responses to specific therapies. The clinical behavior of basal-like and HER2+ subtypes is more aggressive than that of luminal tumors, whereas the luminal A subtype corresponds to low-proliferating tumors with a good prognosis factor [2].

MicroRNAs (miRs) are evolutionary conserved, small non-coding RNA molecules consisting of approximately 22 nucleotides. MiRs inhibit the gene expression post-transcriptionally by binding specifically to the 3′untranslated-region (UTR) of their target mRNA. This gene silencing can occur through translational inhibition of the mRNAs or their cleavage depending on the complementarity between binding site sequences of the target mRNA and miR [3]. Computational analyses indicate that one miR has binding affinity to hundreds of different mRNAs and hence, miRs are involved in the regulation of various cellular processes, such as development, differentiation and proliferation [4]. As miR loci frequently map to fragile chromosomal regions harboring DNA amplifications, deletions or translocations, their expression is often deregulated during tumorigenesis, contributing to tumor progression [5]. In this context, they may act as so-called oncomiRs by targeting tumor suppressor genes or as tumor suppressor miRs by inhibiting oncogenes [6]. MiRs are released into the blood circulation by apoptotic and necrotic cells or by active secretion in small particles [7], and thus, exist either cell-free, associated with proteins or in exosomes in blood [8]. Exosomes are membrane-derived vesicles and may be important mediators of intercellular communication. They transfer lipids, proteins, mRNAs and miRs from one cell to another cell [9]. Thereby, tumor cell-derived exosomes have an emerging role in tumor progression and metastases [10].

To date numerous miRs have been identified, those transcript levels were dysregulated in the blood of breast cancer patients [11, 12]. However, little is known whether dysregulated miR expression levels reflect the distinct molecular subtypes of breast cancer [12]. In our recent study we detected that increased concentrations of miR-373 in postoperative serum of breast cancer patients were associated with negative HER2 status of the primary tumor [13]. This interesting finding provoked us to analyze the miR-373 cluster, consisting of miR-371, miR-372 and miR-373, in particular. The cluster is specifically expressed in human embryonic stem cells (ESCs) and frequently upregulated in several tumors. A role of miR-371 as potential serum biomarker for testicular germ cell tumors was reported [14]. Invasion and metastasis of breast cancer may be stimulated by miR-373, at least partly by direct inhibition of protein expression of the cell surface marker CD44 [15]. MiR-372 and -373 were reported to activate the Wnt/β-catenin-signaling pathway, which is involved in both stem cell maintenance and tumorigenesis [16]. We also focused on circulating miR-101, because this miR plays a role in estrogen-independent signaling and is linked with reduced PTEN (phosphatase and tensin homolog) activity leading to Akt activation [17]. MiR-101 belongs to a family of miRs that is involved in a series of cellular activities, such as cell proliferation, invasion and angiogenesis, and is located in a genomic fragile region that is associated with abnormal deletion or amplification in cancer [18, 19].

The aim of our study was to evaluate whether dysregulated levels of circulating miR-101, miR-371, miR-372 and miR-373, cell-free or localized in exosomes, can be detected in the blood serum of breast cancer patients, and whether these changes are associated with the pathogenesis of this cancer entity. Based on the advantages of blood-based “liquid biopsies” over tissue biopsies and the characteristics of these miRs, their screening could provide valuable non-invasive information in the context of distinct molecular subtypes of breast cancer.

## RESULTS

### Profiling of cell-free miR-101, miR-371, miR-372 and miR-373 in the serum of patients with breast cancer or benign breast disease

**Figure 1:**
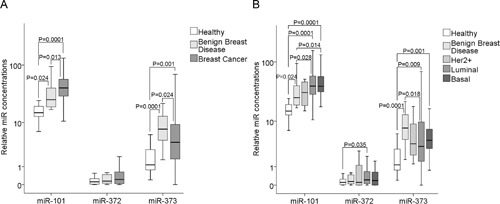
Quantification of cell-free miR-101, miR-372 and miR-373 in the serum of patients with invasive breast cancer and benign breast diseases, and healthy women **(A)** The box plot compares the miR concentrations in the serum of healthy women (n=28) with patients with benign breast disease (n=19) and breast cancer patients (n=168). **(B)** Additionally, the concentrations of miR-101, miR-372 and miR-373 in the serum of healthy women (n=28) and patients with benign breast disease (n=19) were compared with the luminal (n=119), HER2^+^ (n=5) and triple negative (n=33) subtype. The relative expression levels were determined by the low cycle threshold (Ct) values. As determined by the Mann-Whitney-U test, the significant p-values of the statistical evaluations are indicated.

We quantified the relative levels of cell-free miR-101, miR-371, miR-372 and miR-373 in the serum of 168 patients with invasive breast cancer before surgery, 19 patients with benign disease and 28 healthy women. As shown in the box plot of Figure 1A, the serum levels of cell-free miR-101 could significantly differentiate between breast cancer and benign breast disease (p=0.013). There was a continuous rise of serum levels of miR-101 from healthy women over patients with benign breast disease (p=0.024) to patients with breast cancer (p=0.0001). It was surprising to detect the different regulation of miR-371, miR-372 and miR-373 that are located in the same cluster on the chromosomal region of 19q13.42. The miR-371 levels were too low to quantify them (data not shown), and the concentrations of cell-free miR-372 were not dysregulated in patients with breast cancer or benign diseases, whereas the concentrations of miR-373 increased in both (benign, p=0.0001 and malignant, p=0.001) patient cohorts compared with healthy individuals. In contrast to serum miR-101, the serum levels of miR-373 were significantly higher in patients with benign breast diseases than in patients with breast cancer (p=0.024), but could, consequently, differentiate between these two patient cohorts (Figure 1A).

We divided our cohort of 168 patients into the molecular subtypes of breast cancer: 60 luminal A (ER^+^ and/or PR^+^, HER2^−^, Ki-67 low), 59 luminal B (ER^+^ and/or PR^+^, HER2^−^ and Ki-67 high or HER2^+^ and Ki-67 low/high), 33 triple negative (ER^−^, PR^−^, HER2^−^) and 5 HER2^+^ (ER^−^, PR^−^, HER2^+^) patients. For statistical reasons, we combined the luminal A and B subtype. In the subtypes, the serum levels of miR-101 and miR-373 were often significantly higher than the levels in patients with benign breast diseases or healthy individuals, but they did not differentiate between the subtypes. With the exception of luminal tumors (p=0.035), the concentrations of cell-free miR-372 were not dysregulated in the subtypes (Figure 1B).

In addition, we compared the concentrations of cell-free miR-101, miR-372 and miR-373 with the clinical and histopathological risk factors. Supplementary table 1 summarizes the median, mean and p values, as well as the 95 percentiles of the miR variables in the different patient subgroups. We detected that the serum levels of cell-free miR-101 significantly correlated with negative lymph node status (p=0.008, Supplementary figure 1), indicating that a decrease in its concentration plays a role in lymph-node positive cancer and an increase plays rather a role in non-metastatic cancer. No further correlations with the clinicopathological parameters were detected.

### Profiling of exosomal miR-101, miR-372 and miR-373 in the serum of patients with breast cancer and comparison between cell-free and exosomal miRs

**Figure 2:**
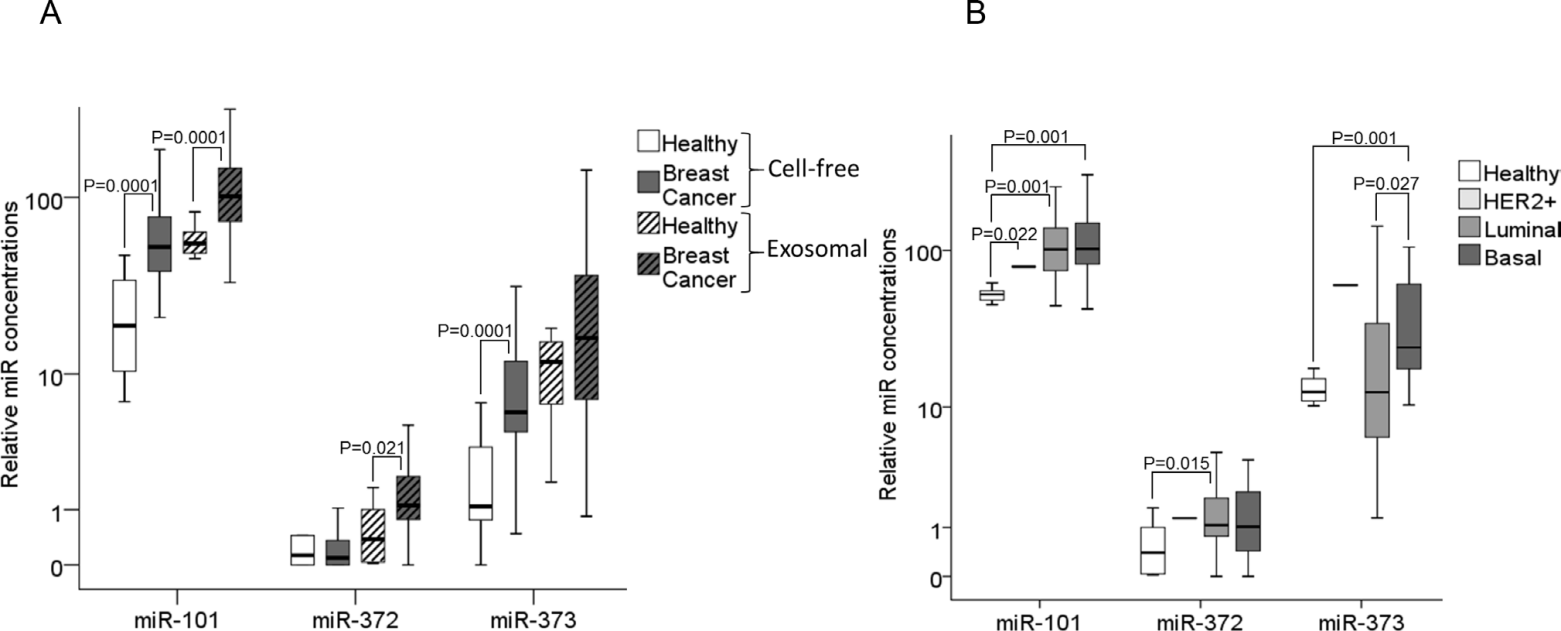
Quantification of cell-free and exosomal miR-101, miR-372 and miR-373 in the serum of patients with invasive breast cancer and healthy women **(A)** The box plot compares the relative values of cell-free and exosomal miR-101, miR-372 and miR-373 in the serum of healthy women (n=12) and breast cancer patients (n=50). **(B)** Additionally, the concentrations of exosomal miR-101, miR-372 and miR-373 in the serum of healthy women (n=12) were compared with the luminal (n=29), HER2^+^ (n=3) and triple negative (n=12) subtype. The statistically significant p-values, as determined by Mann-Whitney-U test, are indicated.

For the additional extraction of exosomes and analyses of exosomal miRs, sufficient amounts of serum were only available from 50 breast cancer patients and 12 healthy individuals within our cohorts (Supplementary table 2). We compared the levels of cell-free and exosomal miR-101, miR-372 and miR-373 in the serum of these cancer patients with those of healthy women. In both cohorts the relative serum concentrations of the exosomal miRs were usually higher than those of the cell-free miRs (Figure 2A). This increase in exosomal miRs can be explained by the fact that the raw CT values of miR-101, miR-372 and miR-373 were similar between serum and exosomes. However, the CT values of the endogenous control miRs (miR-16 and miR-484) were higher in the exosomes than in the serum, whereas the values of healthy controls and breast cancer patients were similar in each fraction (see Material and Methods). As a result the normalization led to higher relative values of miR-101, miR-372 and miR-373 in the exosomal fraction than in the serum. Hence, our findings point to a predominant circulation of miR-101, miR-372 and miR-373 in exosomes in the bloodstream. Moreover, the levels of exosomal miR-101 (p=0.0001) and miR-372 (p=0.021) were tumor-specifically increased, thus, higher in breast cancer patients than healthy controls, whereas the levels of exosomal miR-373 showed no significant difference between breast cancer patients and healthy women (Figure 2A).

**Figure 3:**
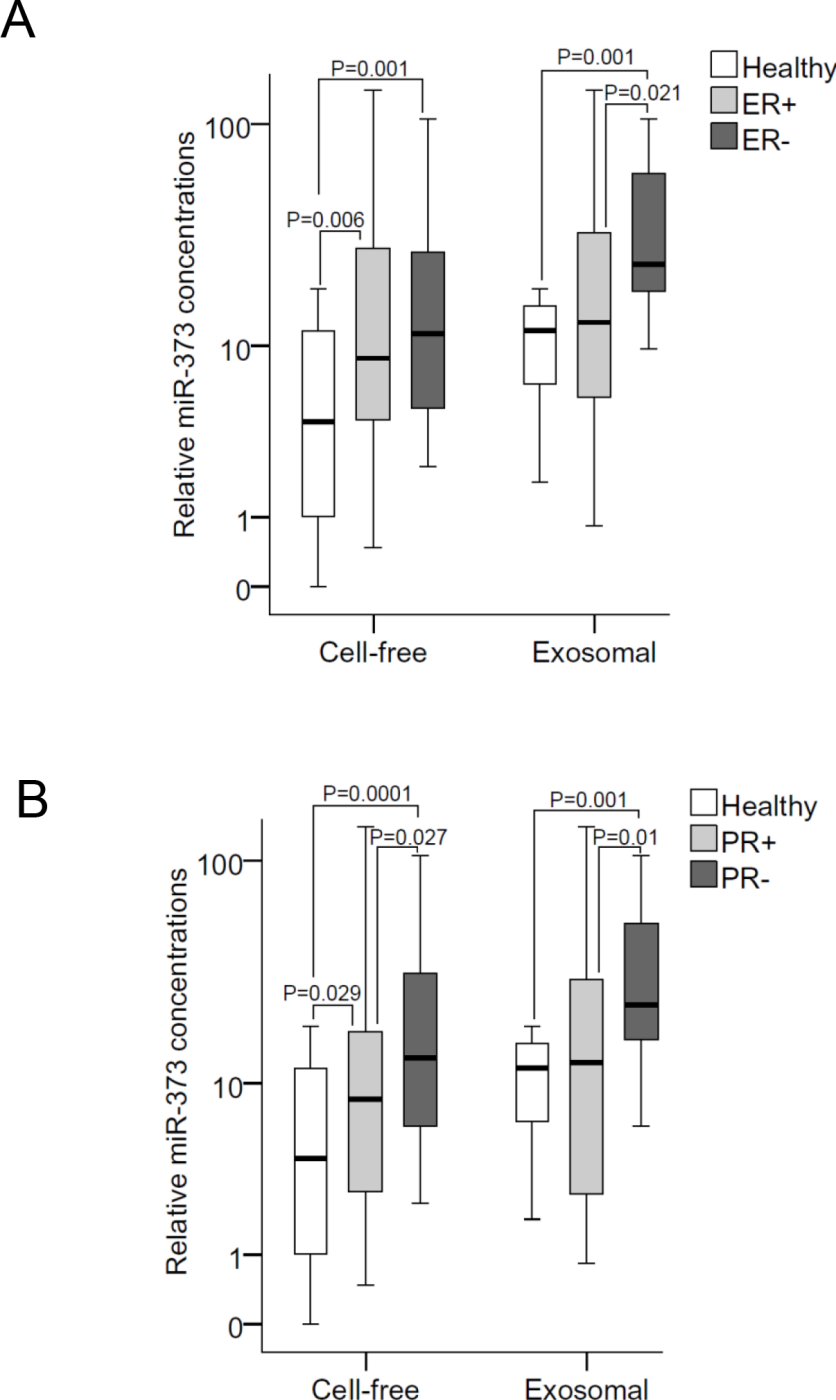
Quantification of cell-free and exosomal miR-373 in the serum of patients with different hormone receptor statuses **(A)** The box plot compares the relative values of exosomal miR-373 in the serum of healthy individuals (n=12) with ER^+^ (n=35) and ER^−^ (n=14) patients; and **(B)** with PR^+^ (n=28) and PR^−^ (n=22) patients. The statistically significant p-values, as determined by Mann-Whitney-U test, are indicated.

In all three molecular subtypes (HER2^+^, p=0.022; luminal, p=0.001; triple negative, p=0.001), the serum levels of exosomal miR-101 were dysregulated compared with the levels in healthy women. Again, with the exception of luminal tumors (p=0.015), the concentrations of exosomal miR-372 were not dysregulated in the subtypes. The serum levels of exosomal (but not of cell-free) miR-373 were significantly higher in triple negative than in luminal breast cancer (p=0.027) and healthy women (p=0.001, Figure 2B). Likewise, the serum levels of exosomal miR-373 were also higher in ER^−^ (p=0.021, Figure 3A) and PR^−^ (p=0.013, Figure 3B) breast cancer patients than the analogous receptor-positive patients, indicating the association of exosomal miR-373 with negative receptor status.

Furthermore, we compared the concentrations of exosomal miR-101, miR-372 and miR-373 with the other clinical and histopathological risk factors und found no significant correlations (Supplementary table 2).

### MiR-373 affects the protein expression of estrogen receptor

Since our findings suggest that miR-373 is associated with receptor-negative breast cancer, we examined whether ER or PR are targets of miR-373. Therefore, we screened the miR databases DIANA-microT-CDS [20], microRNA.org [21] and TargetScanHuman [22] for potential binding sites of miR-373 in the 3′UTRs of ER and PR. We only detected a potential binding site in the 3′UTR of ER, but not in PR.

**Figure 4:**
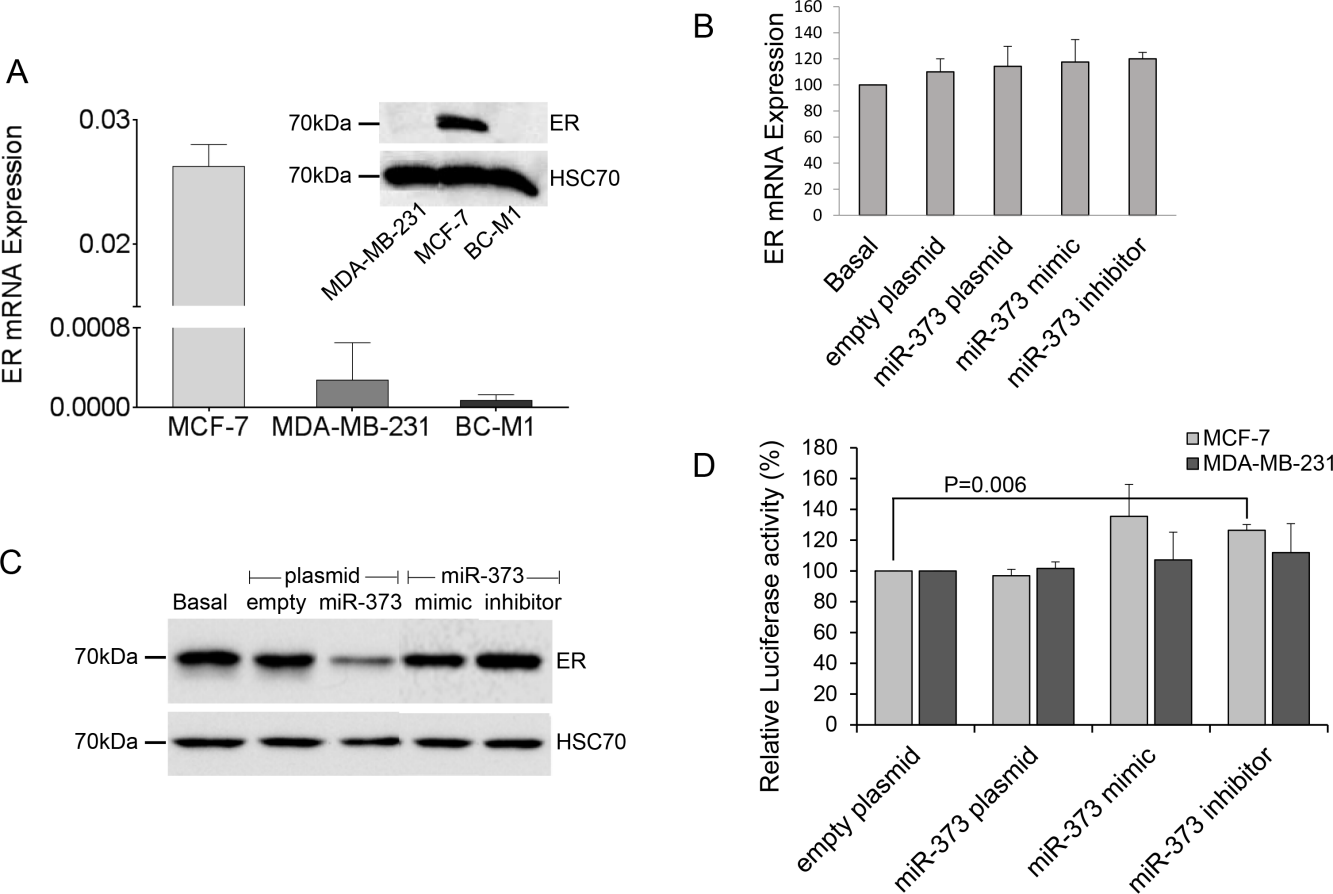
Downregulation of ER protein levels by miR-373 **(A)** Comparison of the relative expression of ER mRNA (bar chart) and ER protein (Western blot) in non-invasive MCF-7 cells with those in invasive MDA-MB-231 cells and micrometastatic BC-M1 cells. **(B)** ER mRNA levels in MCF-7 cells were quantified by real-time PCR. The relative mRNA expression levels were determined by the low cycle threshold (Ct) values. **(C)** ER protein levels in MCF-7 cells were analyzed by Western blot. HSC70 signals served as a loading control. ER mRNA (B) and protein levels (C) in cells non-transfected (basal), transfected with the empty expression plasmid or plasmid encoding for miR-373, with mimics or inhibitors of miR-373. **(D)** Luciferase activity of the reporter plasmid containing the 3′UTR of ER with three binding sites of miR-373 in MCF-7 and MDA-MB-231 cells which were transiently co-transfected with the empty expression plasmid or plasmid encoding for miR-373, with mimics or inhibitors of miR-373. The basal activity was set to 100%. The activities derived from the reference plasmid encoding for the Renilla luciferase were used to normalize the variability in transfection efficiency. The significant p-values as determined by the one-way ANOVA test and standard deviations from triplicate experiments are indicated.

To examine, whether the expression of ER is downregulated by miR-373, we first investigated the expression levels of ER in cell lines MCF-7, MDA-MB-231 and micrometastatic breast cancer cells BC-M1. We found high RNA and protein expression levels of ER in MCF-7 cells, whereas MDA-MB-231 and BC-M1 cells only expressed low levels of ER mRNA, but no protein (Figure 4A). Therefore, only MCF-7 cells were transiently transfected with miR-373 mimic or an expression plasmid encoding for miR-373. The mimics are double-stranded RNA molecules which mimic the mature endogenous miR-373. We confirmed the overexpression of miR-373 in the transfected cells by real-time PCR (data not shown). The real-time PCR data showed no change in ER mRNA levels through an overexpression of miR-373 (Figure. 4B). However, as shown in Western blot analysis using an ER-specific antibody, overexpression of miR-373 could downregulate the protein levels of ER in MCF-7 cells. However, the transfected expression plasmid encoding for miR-373 had a stronger inhibitory effect on ER protein expression than the transfected miR-373 mimic (Figure 4C). Moreover, we also transfected MCF-7 cells with a single stranded miScript miRNA Inhibitor hsa-miR-373 that has binding affinity to the endogenous miR-373. In contrast to the above data, we did not observe any effect on ER protein expression. This discrepancy may be explained by a rather low miR-373 expression level (Ct of 35.52 with standard deviation of 0.38) in MCF-7 cells that can be hardly influenced by the exogenous inhibitor (Figure 4C).

To verify the inhibitory effect of miR-373 on ER protein expression, we carried out a luciferase assay. We transiently co-transfected a reporter plasmid containing the 3′UTR of ER with three binding sites of miR-373 with an expression plasmid of miR-373 or miR-373 mimic or inhibitor into MCF-7 and MDA-MB-231 cells, and measured the luciferase activity. Ectopic expression of miR-373 did not decrease the luciferase activity. However, inhibition of miR-373 increased the activity in MCF-7 cells (p=0.006), but not in MB-MDA-231 cells (Figure 4D).

These somewhat discrepant results between Western blot and luciferase assay cannot be explained, but they suggest that miR-373 is involved in the repression of ER protein expression by inhibiting the translation of its mRNA.

### MiR-373 inhibits camptothecin induced apoptosis

**Figure 5:**
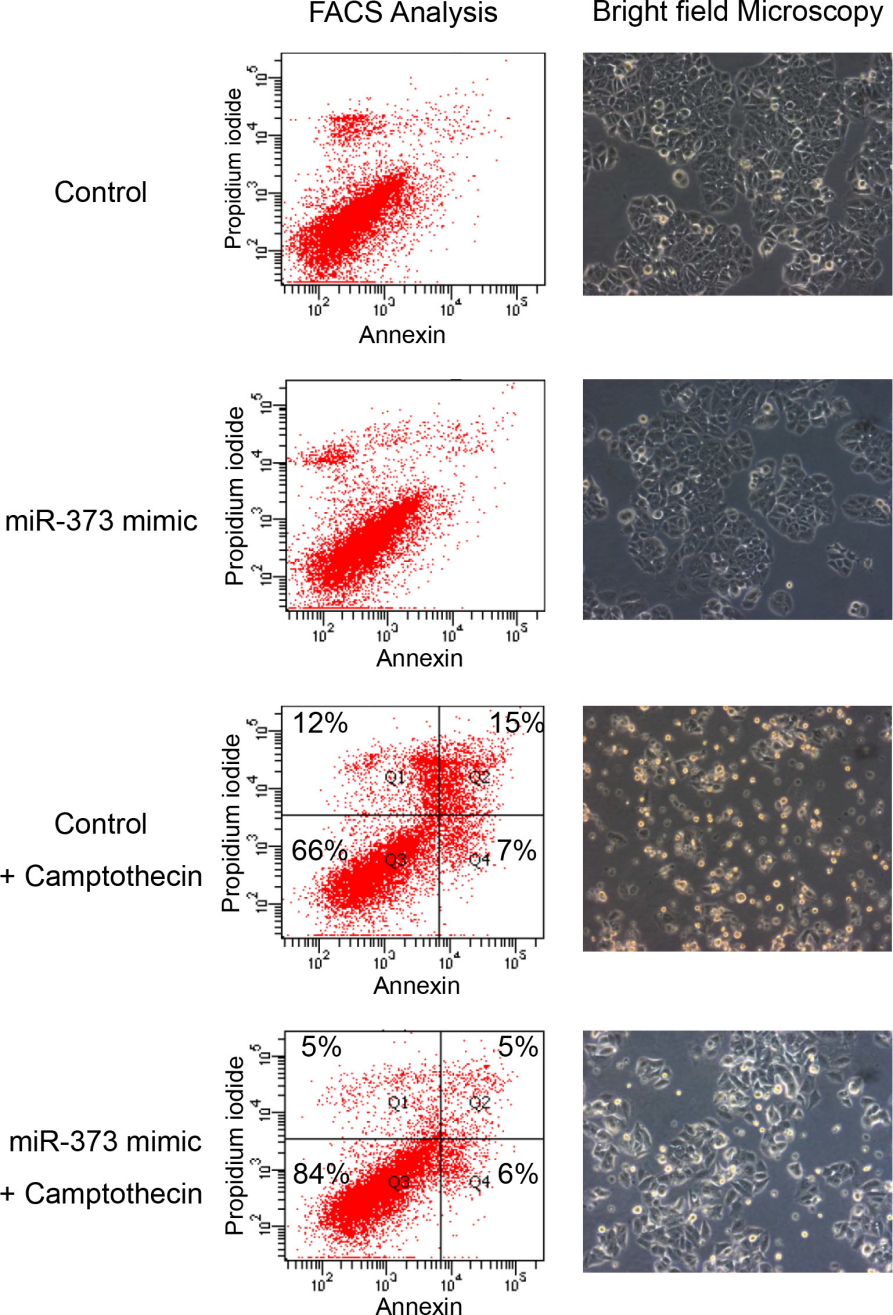
Inhibition of camptothecin mediated apoptosis by miR-373 overexpression MCF-7 cells transfected with miR-373 mimic and treated with the topoisomerase I inhibitor camptothecin were analyzed on a FACS CantoII device (left) and by a bright field microscopy (right). A 10x magnification of the Axiovert 200 microscope was performed. Cells were labeled with Annexin-V-FITC and propidium iodide for FACS analyses. Cell fragments only positive for propidium iodide can be found in the upper left corner (Q1). Late apoptotic as well as necrotic cells can be found in the upper right corner (Q2), since they are positive for Annexin and propidium iodide. Living cells are negative for Annexin and propidium iodide, and therefore, can be found in the lower left corner (Q3). Only early apoptotic cells are positive for Annexin, and located in the lower right corner (Q4). The size for each population (%) is given in the corresponding area.

The effect of miR-373 on apoptosis was also investigated in MCF-7 cells transfected with miR-373 and treated with the topoisomerase I inhibitor camptothecin. Camptothecin, which is used in cancer chemotherapy, induces apoptosis. Brightfield images and FACS analyses showed an inhibitory effect on camptothecin mediated apoptosis by miR-373 overexpression in MCF-7 cells (Figure 5). However, we did not observe any effect on apoptosis by the miR-373 inhibitor (data not shown). Additionally, the effects of miR-373 on cell proliferation were evaluated by a MTT assay. The viable cell number did not change by the transfection of MCF-7 and MDA-MB-231 cells with an expression plasmid encoding for miR-373, or miR-373 mimic or inhibitor, suggesting that miR-373 has no effect on cell proliferation (Supplementary figure 2).

### MiR-101 has no effect on the migration and invasion of MCF-7 and MDA-MB-231 cells

Our current findings show that low levels of miR-101 correlate with a positive lymph node status. To examine whether this observation can be explained by an effect of miR-101 on cell migration and invasion, respective assays were carried out using MCF-7 and MDA-MB-231 cells. We, therefore, used these two cell lines, because MCF-7 and MDA-MB-231 cells display low and high migration/invasion potential, respectively. Using transwell assays, we couldn't observe any change in cell migration or invasion after transfection of MCF-7 and MDA-MB-231 cells by miR-101 mimic or inhibitor (Supplementary figure 3).

## DISCUSSION

In the present study we demonstrate that in breast cancer patients and healthy women the relative exosomal serum concentrations of miR-101, miR-372 and miR-373 were usually higher than their cell-free levels, indicating that these miRs may predominantly circulate in exosomes in the peripheral blood. Furthermore, the serum levels of cell-free miR-101 and miR-373 could significantly differentiate between breast cancer and benign breast disease, indicating their potential diagnostic value. The reasons for the high miR-373 levels in the serum of patients with benign breast disease are unclear, but could be explained among others by the fact that benign lesions reflect inflammatory processes resulting in elevated apoptotic cell death. Our recent report [23] showed that the levels of miR-373 were still increased after surgery which might indicate inflammation. In contrast to the increased concentrations of cell-free miR-101 in tumor patients compared with healthy women, we detected that the serum levels of miR-101 negatively correlated with lymph node status. Our findings suggest that decreased miR-101 concentrations are associated with lymph-node positive cancer and an increase plays rather a role in non-advanced cancer. Although miR-371, miR-372 and miR-373 are localized in the same cluster, miR-371 was not detectable in serum, and miR-372 was not dysregulated in benign and malignant tumors. The levels of exosomal miR-101 and miR-372 were tumor-specifically increased and higher in breast cancer patients than in healthy women. Whereas a tumor-specific increase for both, cell-free and exosomal miR-101 was observed, the data on cell-free and exosomal miR-372 and miR-373 were converse. The levels of exosomal (but not of cell-free) miR-372 and of cell-free (but not of exosomal) miR-373 increased in breast cancer patients. In particular, the serum levels of exosomal (but not of cell-free) miR-373 were significantly higher in triple negative than in luminal breast cancer. Likewise, the serum levels of exosomal miR-373 were also higher in ER^−^ and PR^−^ breast cancer patients than in the corresponding receptor-positive patients, indicating the association of exosomal miR-373 with negative receptor status. Our functional analyses showed that miR-373 may downregulate the protein expression of ER and inhibit apoptosis induced by camptothecin.

As far as we know, our present article is the first study reporting the comparison between cell-free and exosomal miRs in breast cancer patients and healthy women. Usually, we detected the prevalence of miR-101, miR-372 and miR-373 in the exosomal serum fraction of breast cancer patients and healthy women. Whether miRs exist cell-freely in the blood circulation or are transported by secreted exosomes has been discussed [24]. Our present data support both possibilities but with a predominant circulation of miRs in exosomes. The high miR concentrations observed in exosomes may be dependent on that exosomes protect miRs from digestion by RNases and, therefore, exosomal miRs may circulate more stable in blood than cell-free, unbound miRs.

A number of published studies have reported that miR-101 is obviously down-regulated and negatively associated with tumor growth and blood vessel formation in several solid epithelial cancer types, e.g., glioblastoma, non-small cell lung cancer or ovarian cancer [18, 25, 26]. In contrast to the published data, our findings show a cancer-specific increase in serum miR-101 in breast cancer patients compared with patient with benign breast disease and healthy women, but a decrease in lymph node-negative breast cancer, suggesting a dual role for miR-101 in breast cancer. A dual role was also described for miR-101 in the context of estradiol (E2)-independent and -dependent growth. Although miR-101 inhibited cell growth in normal E2-containing medium, it promoted cell growth in E2-free medium. Moreover, estrogen deprivation significantly enhanced miR-101-mediated activation of the Akt signaling pathway [17], which gives rise to increased cell survival, malignant transformation, invasiveness and metastasis of breast cancer [27]. Similar to our data showing that the serum levels of cell-free miR-101 were significantly higher in patients with breast cancer than in patients with benign breast disease, the expression of miR-101 was also significantly higher in another cancer type, in malignant pheochromocytoma, as compared with its benign counterpart [28]. Besides, lower expression of miR-101 was associated with poor prognosis of patients with endometrial serous adenocarcinomas [29] and might endure our observation on the decreased serum levels of miR-101 in lymph-node positive breast cancer patients. The association of miR-101 with lymph node status does not seem to be caused by a change in cell invasion or migration through miR-101, as shown by our functional analyses.

For miR-372 and miR-373, different results regarding their cell-free or exosomal concentrations in breast cancer patients and healthy women were found. To explain this difference it is necessary to further examine the exosomal fraction and to distinguish between tumor-derived and wild type-derived exosomes. In this regard, Rupp et al. reported that breast cancer patients have increased amounts of exosomes but that there is still a lack for a tumor-exosomal marker protein [30].

In respect to serum miR-373, the levels of exosomal miR-373 were associated with negative ER and PR status. The serum concentrations were higher in ER^−^ and PR^−^ patients than in patients with positive receptor status, and higher in triple negative than in luminal breast carcinomas. Our findings point to the ability of miR-373 to downregulate these receptors. However, a screening of different miR databases showed that with the exception of ER, the other two receptors PR and HER2 are not predicted to be targeted by miR-373. In this context, our functional analyses showed that miR-373 was able to downregulate the protein expression of ER. This downregulation, which was also demonstrated in a previous study [31], may be linked to increased transcript levels of miR-373 in ER^−^ breast cancer. Surprisingly, the levels of cell-free miR-373 did not reflect the increase in exosomal miR-373 in receptor-negative tumors. Based on the clinical behavior of the basal-like breast cancer subtype, including triple negative tumors [2], exosomal miR-373 may be associated with more aggressive tumors. This assumption is also supported by our transfection studies. Modulation of miR-373 expression in MCF-7 cells revealed that this miR inhibited apoptosis of MCF-7 cells, which was induced by camptothecin, but had no effect on cell proliferation. In contrast to breast cancer cells, a previous report showed that miR-373 could increase the cell proliferation of esophageal cancer cells [32]. As far as we know, our study demonstrates, for the first time, that miR-373 is involved in the apoptosis mediated by camptothecin but further studies are necessary to analyze the proteins targeted by miR-373 in this process. Finally, it has been recently reported that miR-373 may be a potential plasma-based biomarker for detecting the lymph node status of breast cancer [33]. However, we could not detect such an association of serum miR-373 with lymph node metastases in our study cohort.

In conclusion, our findings suggest cell-free miR-101 and miR-373 as breast cancer-specific markers. Moreover, the association of increased exosomal serum levels of miR-373 with receptor-negative tumors and its anti-apoptotic character show the potential role of exosomal miR-373 as a blood-based biomarker for more aggressive tumors. Prospective studies on larger cohorts of patients are required to substantiate the diagnostic role of exosomal miRs in comparison with cell-free miRs.

## MATERIALS AND METHODS

### Study populations

Serum samples from 168 patients with primary breast cancer before surgery and chemotherapy and 19 patients with benign breast disease (10 patients with fibroadenoma, 8 patients with mastopathy and 1 patient with papilloma) were obtained from the Clinic of Gynecology of the University Medical Center Hamburg-Eppendorf. In addition, serum samples were collected from 28 age-matched healthy women with no history of cancer and in good health based on self-report.

Women were eligible for inclusion in this project, if they had histologically confirmed primary invasive breast cancer (International Classification of Diseases) and diagnosed between January 2001 and September 2005. The study was approved by the ethic committee of the Medical Board of Hamburg, and conducted in accordance with the Declaration of Helsinki. All study participants gave written informed consent.

Tumors were histologically categorized as ductal, lobular and for statistical reasons as all others (1 invasive mucinous, 3 invasive papillar, 2 invasive ductal-mucinous, 4 invasive tubular, 4 invasive medullar breast cancer and 5 unknown patients). Grading of all invasive carcinomas was based on a modification of Bloom and Richardson, recommended by Elston and Ellis [34]. Hormone receptor status was determined using immunohistochemical assays and immunoreactive scoring according to Remmele and Stegner [35]. HER2 expression was assessed using standardized immunohistochemical analyses. In case of an ambiguous positive reaction (DAKO score 2+), these analyses were followed by a fluorescent in situ hybridization test. The clinicopathological data of the patients are listed in Table 1.

**Table 1:** Patient characteristics at the time of primary diagnosis of breast cancer

Parameters	n (%)
Breast cancer patients	
**Total**	168
**Age**	59 years (range 30-85 years)
**Histology**	
ductal	109 (65)
ductu-lobular	9 (5)
lobular	31 (19)
others	19 (11)
**Tumor stage**	
pT1	93 (56)
pT2-4	74 (44)
**Lymph node metastasis**	
neg.	114 (68)
pos.	53 (32)
**Grading**	
1,2	98 (60)
3	64 (40)
**Estrogen receptor status**	
negative	38 (23)
positive	127 (77)
**Progesterone receptor status**	
negative	68 (41)
positive	98 (59)
**HER2 status**	
negative	129 (82)
positive	28 (18)
**Subtype**	
luminal	119 (76)
HER2 positive	5 (3)
Triple negative	33 (21)
**Overall survival**	
alive	147 (90)
dead	16 (10)
**Disease-free survival**	
Recurrence-free	147 (90)
Recurrence	16 (10)

### Extraction of total RNA

Extraction of total RNA from human blood serum was performed by the mirVana PARIS kit (Life Technologies, New York, USA) according to the manufacturer's instructions. Four hundred μL of serum were incubated with an equal volume of Denaturation Solution for 5 min. on ice. For RNA extraction acid-phenol:chloroform was used, and precipitation was carried out by ethanol and a filter cartridge. The RNA was eluted in 100 μL of preheated Elution Solution and quantified on a NanoDrop ND-1000 Spectrophotometer (Thermo Scientific, Wilmington, Delaware, USA). The RNA samples were immediately stored at −80°C until they were reverse transcribed into cDNA.

### Isolation of exosomes

Exosomes were isolated by the ExoQuick exosome precipitation solution (BioCat, Heidelberg, Germany) according to the manufacturer's instructions. Four hundred μL of serum were incubated overnight, on ice, with 100 μl of the ExoQuick solution. Following precipitation of exosomes, exosomal RNA was extracted using the mirVana PARIS kit (Life Technologies) and exosomal protein was extracted using RIPA (150mM NaCl, 1% NP-40, 0.5% sodium-deoxycholate, 0.1% SDS, 50mM Tris/HCl, pH 8) buffer. The extraction of exosomes was verified by Western Blot using an antibody specific for the exosomal marker proteins CD63 (BioCat), Mucin1 (CD227, BD Biosciences) and GAPDH (Sigma, Supplementary figure 4).

### Conversion of total RNA into cDNA

Reverse transcription was performed by the TaqMan MicroRNA Reverse Transcription Kit (Life Technologies) briefly after RNA extraction. The 10 μL-reverse transcription reaction contained 0.1 μL 100 mM dNTPs, 0.66 μL MultiScribe Reverse Transcriptase (50 U/μL), 1 μL 10× Reverse Transcription Buffer, 0.13 μL RNase Inhibitor (20 U/μL), 2 μL 5x TaqMan RT Primer, nuclease-free water and 4 μL RNA. The reaction was carried out at 16°C for 30 min., 42°C for 30 min. and 85°C for 5 min. on a MJ Research PTC-200 Peltier Thermal Cycler (Global Medical Instrumentation, Ramsey, Minnesota, USA). The cDNA samples were stored at −20°C until further usage.

### Preamplification of miR-371, miR-372, miR-373, miR-16 and miR-484 cDNA

Due to the low expression levels of miR-371, miR-372 and miR-373 in healthy women, a preamplification of its cDNA was performed. For an accurate normalization, cDNA of the reference miR-16 and miR-484 was also preamplified. The cDNAs were preamplified in 5μL Taq PCR Mastermix, 0.5 μL 20x TaqMan MiRNA Assay mix and nuclease-free water by using the Taq PCR Mastermix Kit (Qiagen, Hilden, Germany). The PCR was run on an MJ Research PTC-200 Peltier Thermal Cycler (Global Medical Instrumentation): 1 cycle at 95°C for 5 min., 15 cycles at 95°C for 20 s, 60°C for 20 s and 72°C for 20 s and a terminal cycle at 72°C for 5 min.

### Quantitative real-time PCR of miR-101, miR-371, miR-372 and miR-373

For quantitative real-time PCR, the miR-specific TaqMan MicroRNA Assays (Life Technologies) for miR-16 and miR-484 (reference miRs), miR-101, miR-371, miR-372 and miR-373 (candidate miRs) were used. MiR-16 and miR-484 were, therefore, used as reference miRs, because they were constantly expressed in our study populations and recommended as reference miRs in the literature [36, 37]. Despite other reports miR-16 was equally expressed in healthy women, patients with a benign breast disease and breast cancer patients, as shown by the raw CT-values given below. For the normalization of RNA extraction and cDNA synthesis 20 fmol of synthetic cel-39 was added to the serum samples after addition of the denaturation solution. In a 10 μL-reaction, 1 μL cDNA were mixed with 5 μL TaqMan Universal PCR Master Mix No AmpErase UNG and 0.5 μL miR-specific TaqMan MicroRNA Assay Mix on a twin-tec real-time PCR plate (Eppendorf, Hamburg, Germany). The quantitative real-time PCR reaction was performed at 95°C for 10 min. and in 40 cycles at 95°C for 15 s and 60°C for 60 s on a Mastercycler Realplex (Eppendorf).

The obtained data of the miR expression levels were calculated and evaluated by the ΔCt method as follows: ΔCt = mean value Ct (mean of reference miR-16, miR-484 and cel-39) - mean value Ct (miR of interest). The relative expression of the miR of interest corresponded to the 2^(ΔCt) value.

To verify whether the normalization of the expression data of miR-101, miR-371, miR-372 and miR-373 by miR-16 and miR-484 was accurate, we analyzed the miR-16 and miR-484 expression levels, and found that the levels remained relatively constant across the serum samples. We calculated a mean value of both miRs of 26.8, 27.3 and 27.2 with a standard deviation of 1.4, 1.7 and 1.4 for the groups of patients with breast cancer, patients with benign breast disease and healthy women, respectively. For the expression of exosomal miR-16 and miR-484 we calculated a mean value of both miRs of 28.9 and 28.6 with a standard deviation of 1.1 and 1.3 for the patients with breast cancer and healthy women, respectively.

### Cell culture

The breast adenocarcinoma cell lines MCF-7 and MDA-MB-231 were cultured in DMEM (Life Technologies) supplemented with 10% FCS (fetal calf serum; PAA Laboratories, Cölbe, Germany) and 2 mM L-glutamin (Life Technologies) under standard conditions (37°C, 10% CO_2_, humidified atmosphere). The micrometastatic BC-M1 (breast cancer) cells were cultured at 37°C, 5% CO2 and 10% O2 in RPMI (Invitrogen, Karlsruhe, Germany) supplemented with 10% FCS (PAA Laboratories), 2 mM L-glutamin (Invitrogen), 10 mg/mL Insulin-Transferrin-Selenium-A (Invitrogen), 50 ng/mL recombinant human epidermal growth factor, and 10 ng/mL human basic fibroblast growth factor (Miltenyl Biotec, Bergisch-Gladbach, Germany). Cell viability was determined by trypan blue staining.

### Transient transfection of miR-373 and miR-101

To analyze whether miR-373 has an influence on the expression of ER, 3*10^5^ of MCF-7 cells were seeded on 6-well plates (NUNC, Roskilde, Denmark) and transfected with 1 μg of an expression plasmid encoding for miR-373 or the double-stranded miScript miRNA Mimic hsa-miR-373 at final concentrations of 5nM (Qiagen) or the single stranded miScript miRNA Inhibitor hsa-miR-373 at final concentrations of 25nM (Qiagen) and with 2 μL X-tremeGENE HP DNA Transfection Reagent (Roche Diagnostics, Mannheim, Germany). After incubation of 48h, total RNA and protein were extracted using peqGOLD TriFast (Peqlab, Erlangen, Germany) according to the manufacturer's instructions. Proteins were quantified by the DC protein assay (Bio-Rad Laboratories) according to the manufacturer's instructions.

The expression plasmid encoding for miR-373 was constructed by annealing the DNA sequences of 5′-TCATACTCGAGATCTGGGATACTCAAAATGGGG GCGCTTTCCTTTTTGTCTGTACTGG-3′ and 5′-AACC GCTCGAGGATCCGGGACACCCCAAAATCGAAGCA CTTCCCAGTACAGACAAAAA-3′. The 5′ overhanging ends were filled in by the Klenow fragment DNA polymerase (Thermo Scientific). The double-stranded DNA was cut by the restriction enzyme XhoI (New England Biolabs, Frankfurt, Germany) and cloned into the XhoI site of the pcDNA 3.1 vector (Life Technologies). The cloned expression plasmid was verified by DNA sequencing.

To analyze whether miR-101 has an influence on the expression of cell migration or invasion, 3*10^5^ of MCF-7 or MDA-MB-231 cells were seeded on 6-well plates (NUNC, Roskilde, Denmark) and transfected with the double-stranded miScript miRNA Mimic hsa-miR-101 at final concentrations of 5nM (Qiagen) or with the single stranded miScript miRNA Inhibitor hsa-miR-101 at final concentrations of 25nM (Qiagen) with 2 μL X-tremeGENE HP DNA Transfection Reagent (Roche Diagnostics, Mannheim, Germany). Transient transfection efficiency was confirmed by real-time PCR of the transfected miR.

### Quantitative real-time PCR and Western Blot of ER expression

To determine the mRNA expression of ER, a total of 200 ng RNA from basal and transfected cells was reverse transcribed using the First strand cDNA synthesis kit (Thermo Scientific). The mRNA expression levels were subsequently quantified by real-time PCR using the Maxima SYBR Green/ROX qPCR Master Mix (Thermo Scientific) and the following primers for ER (forward: 5′-GCATTCTACAGGCCAAATTCA-3′; reverse: 5′-TCCTTGGCAGATTCCATAGC-3′). GAPDH (5′-CCTGCACCACCAACTGCTTAG-3′, 5′-TGGCATGGACTGTGGTCATG-3′) served as reference gene. The reaction was performed at 95°C for 15 min. and in 40 cycles at 95°C for 15 s, 60°C for 30 s and 72°C for 30 s on a Mastercycler Realplex (Eppendorf).

Protein levels of ER in basal and transfected cells were investigated by Western blot analysis. Thirty μg of cell lysates were electrophoretically separated and blotted onto a PVDF membrane (Millipore, Billerica, USA) which was subsequently incubated with antibodies specific for ER (Thermo Scientific) The membrane was reprobed with the anti-Hsc70 antibody (Santa Cruz, Heidelberg, Germany) overnight which served as a loading control. Detection of the proteins was carried out using peroxidase-conjugated secondary antibodies (Dako, Glostrup, Denmark) and the chemiluminescence ECL detection solution (200 μM coumaric acid, 1.25 mM luminol, 0.0009% H_2_O_2_ in 100 mM Tris pH 8.5).

### Luciferase assay

For transient transfection, the 3′UTR of ER containing three miR-373 binding sites (5′-CTAGCTAGAGACCCAGGCCTGGAGAGTAGACA TTTTGCCTCTGATAAGCACTTTTTAAATTTCTAAG TAATTGCTGCCTCTATTATGGCACTTCAATTTTGCA CTT-3′) was cloned into a pCR2.1 TOPO vector (Invitrogen), digested with the restriction enzymes XbaI and NheI (New England Biolabs) and subcloned into the corresponding restriction sites of the pmirGLO Dual-Luciferase miRNA Target plasmid (Promega, Mannheim, Germany). The Luciferase assay was performed using the Dual-Luciferase Reporter Assay (Promega) according to the manufacturer's protocol. Luciferase activity was measured on a 20/20n Luminometer Turner Biosystems (Promega) and normalized by the Renilla luciferase activity. Each transfection experiment was carried out in duplicate wells and repeated two times.

### Apoptosis assay and flow cytometry

To induce apoptosis, 24 hour after transfection MCF-7 cells on a 6-well plate were treated with 4.8 μM of the topoisomerase I inhibitor camptothecin (Biovision, Milpitas, USA) for 4 hours. Twenty-four hours after induction of apoptosis, the non-transfected and transfected cells were incubated with 3 μL Annexin-V-FITC (BD Biosciences, San Jose, CA, USA) and 5 μL propidium iodide (Sigma-Aldrich) in the dark at 4°C for 15 min. Following incubation 400 μL Annexin-FITC binding buffer (0.1 M Hepes pH 7.4, 1.4 M NaCl, 25 mM CaCl_2_ in PBS) was added to the cells. The cells were then analyzed on a FACS CantoII flow cytometer (BD Biosciences). For bright field pictures the Axiovert 200 microscope (Carl Zeiss, Jena, Germany) with a 10x magnification was used.

### Cell migration and invasion assay

Cell migration and invasion were measured using 8-nm pore uncoated or BME (basement membrane extract)-coated transwell inserts, respectively (Trevigen, City of Gaithersburg, USA). Briefly, 8 hours after transfection MCF-7 and MDA-MB-231 cells were grown in starvation media (DMEM with 0.5% FCS and 2 mM L-glutamine) for 24 hours prior to their detachment. Cells were resuspended in 400 μl starvation media and 100,000 cells per well were added to the insert. Medium containing 10% FCS was added to each well, and cells were allowed to migrate and invade for 24 hours at 37°C. Inserts and wells were washed with 1x washing buffer (Trevigen), and migrated or invaded cells were incubated in 500 μl dissociation buffer containing 0.83 μg Calcein-AM (Trevigen) for 1 hour. Fluorescence (excitation: 485 nm, emission: 535 nm) was measured by the LB 940 Mithras microplate reader (Berthold Technologies, Bad Wildbach, Germany).

### MTT assay

To measure cell proliferation, non-transfected and cells transfected with miR-373 expression plasmid, mimic or inhibitor, for 24, 48 and 72 hours were incubated with 20 μL 5 mg/ml MTT (thiazolyl blue tetrazolium bromide, Sigma-Aldrich) in PBS on a 96-well plate at 37°C for 3 hours. Following incubation the cells were lysed with lyses buffer (4 mM HCl, 0.1% NP40 in isopropanol). A microplate reader (Tecan, Männedorf, Switzerland) was used to measure the OD values at 540 nm. Each experimental group contained three replicate wells, and the experiment was repeated three times.

### Statistical analysis

The statistical analyses were performed using the SPSS software package, version 20.0 (SPSS Inc. Chicago, IL). Because of skewed distributions of the miRs concentrations, differences in group levels were bivariately assessed by the Mann-Whitney-U-test. Moreover, statistical differences were calculated using ANOVA with Tukey's HSD test for all pairwise comparisons that correct for experiment-wise error rate. Missing data were handled by pairwise deletion. A p-value <0.05 was considered as statistically significant. All p-values are two-sided. Due to the explorative nature of the study no formal adjustment for multiple testing was performed.

## SUPPLEMENTARY FIGURES AND TABLES


