# Supplementary material for: Increased serum levels of circulating exosomal microRNA-373 in receptor-negative breast cancer patients
Source: Oncotarget. 2014 Oct 11;5(20):9650–63. doi: 10.18632/oncotarget.2520 (PMC4259427; doi:10.18632/oncotarget.2520)
Supplement: Supplementary file 1 [file oncotarget-05-9650-s001.pdf]

SUPPLEMENTARY FIGURES AND TABLES

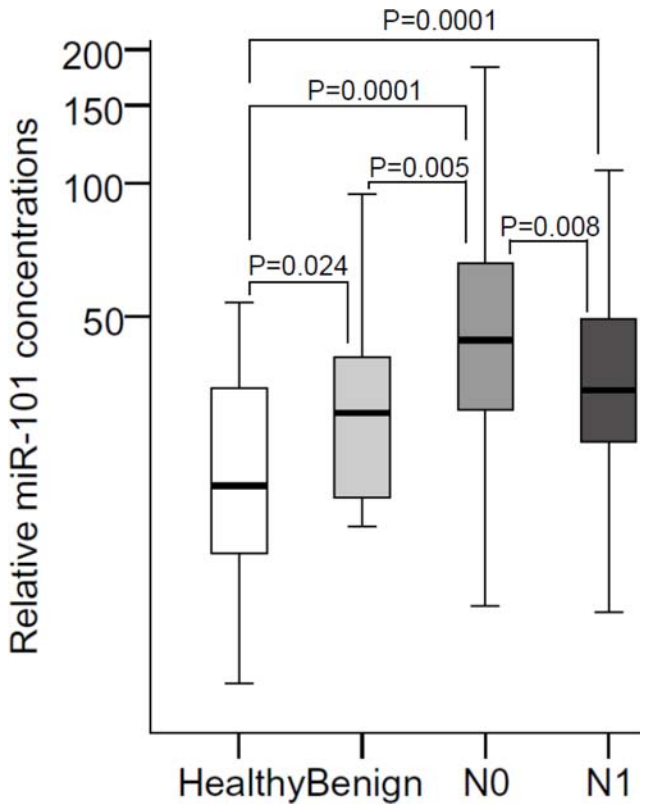

Supplementary Figure S1: Decreased serum levels of cell-free miR-101 are associated with lymph node metastasis. The box plot compares the serum concentrations of miR-101 in lymph node-negative (n=114) with those in lymph node-positive patients (n=53). As determined by the Mann-Whitney-U test, the significant p-values of the statistical evaluations are indicated.

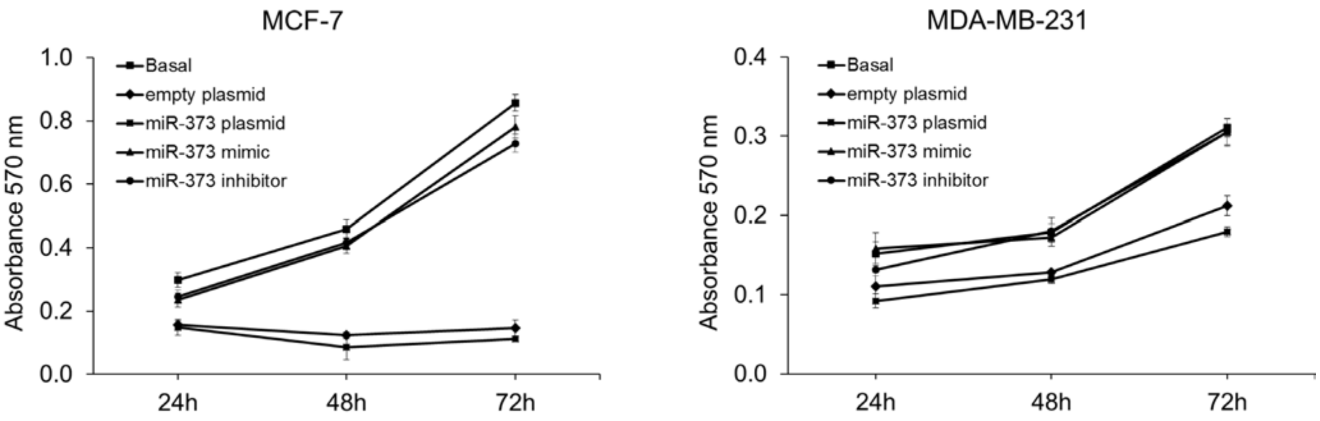

Supplementary Figure S2: miR-373 does not affect cell proliferation. MCF-7 and MDA-MB-231 cells were transiently transfected with empty expression plasmid or plasmid encoding for miR-373, with mimics or inhibitors of miR-373, and treated with MTT. Cell proliferation after overexpression and inhibition of miR-373 for 24, 48 and 72 hours. The standard deviations from triplicate experiments are indicated.

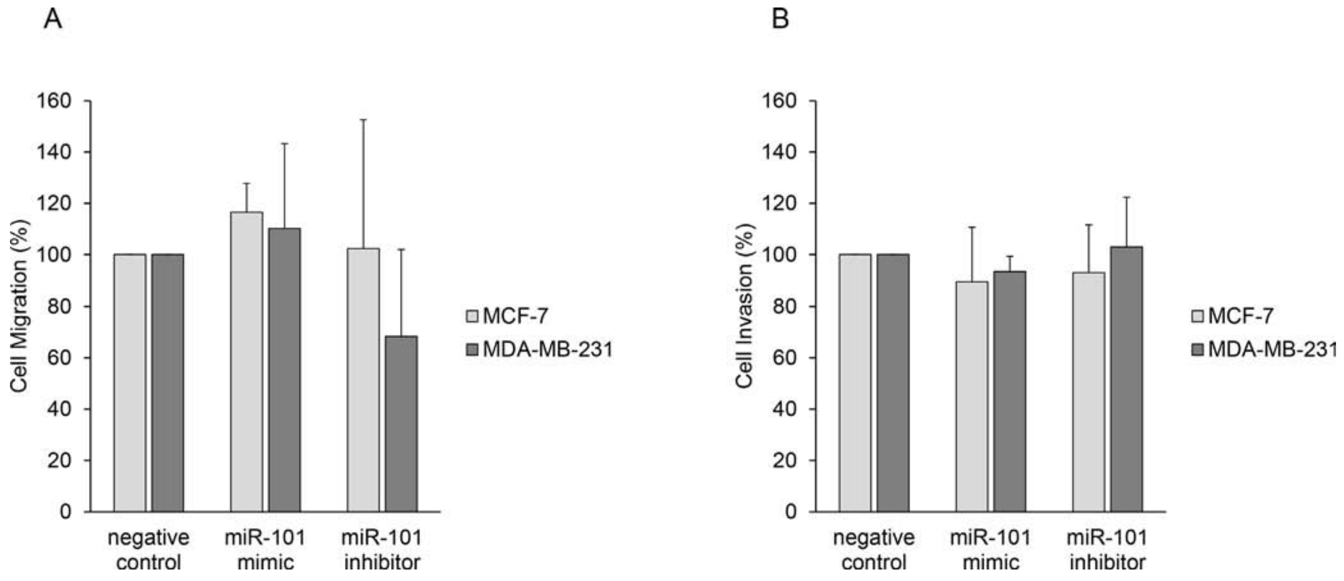

Supplementary Figure S3: miR-101 does not affect cell migration and invasion of breast cancer cells. Cell migration and invasion of MCF-7 and MDA-MB-231 cells were analyzed by transwell assays. Cells were transfected with mimics or inhibitors of miR-101 prior to serum starvation for 24 hours. Cells were added to the upper transwell chamber that was uncoated for the migration assay and BME-coated for the invasion assay, and allowed to migrate or invade for 24 hours. Migrated or invaded cells were stained with Calcein-AM, and fluorescence was measured at excitation of 485nm and emission of 535nm.

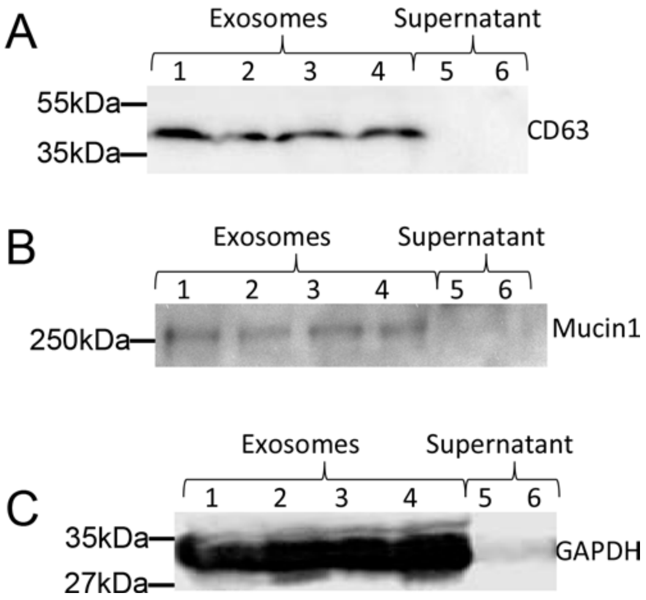

Supplementary Figure S4: Extraction of serum exosomes. Exosomes were extracted and pelleted from serum and analyzed by Western blot using an antibody specific for the proteins **(A)** CD63, **(B)** Mucin1 and **(C)** GAPDH. As a negative control served the supernatant of the pellet that displayed no exosomes when the antibodies against CD63, Mucin1 and GAPDH were added. The Western blot shows representative examples of the exosome extraction from pellets of four breast cancer patients (lane 1, 2, 3, 4) and the negative control of two patients (lane 5, 6).

Supplementary Table S1. Patient characteristics at the time of primary diagnosis of breast cancer and correlation to the relative miR concentrations in serum.

Parameters	n (%)	miR-101			miR-372			miR-373		
		relative values		p values	relative values		p values	relative values		p values
		Median	Mean		Median	Mean		Median	Mean	
		5%	95%		5%	95%		5%	95%	
**Total**		CI			CI			CI		
	168 (100)	42	49		0.2	0.6		3.8	8.4	
		16	106		0	2.6		0.4	43	
**Age**		45 - 54			0.4 - 0.8			5.3 - 10.6		
**Histology**	59 (30-85)									
**ductal**				0.464			0.613			0.934
	109 (65)	41	51		0.2	0.7		3.8	8.4	
		17	118		0	3.1		0.4	40	
**ductu-lobular**		45 - 58			0.4 - 0.9			6.0 - 11		
	9 (5)	48	47		0.2	0.3		2.2	8	
		29	-		0	-		0.7	-	
**lobular**		36 - 58			0.1 - 0.6			0 - 20		
	31 (19)	41	43		0.2	0.4		4.3	10	
		12	93		0	1.8		0.2	73	
**others**		35 - 50			0.2 - 0.5			3.6 - 17		
	19 (11)	43	48		0	0.4		3.2	5.5	
		15	-		0	-		0.3	-	
**Lymph node metastasis**		37 - 59			0.0 - 1.0			1.1 - 9.8		
**neg.**				**0.008**			0.975			0.418
	114 (68)	44	52		0.2	0.6		4.2	9.5	
		18	106		0	3.1		0.4	49	
**pos.**		46 - 57			0.4 - 0.9			6.7 - 12		
	53 (32)	35	41		0.2	0.4		3.3	6.1	
		13	87		0	2.4		0.4	23	
**Tumor stage**		35 - 48			0.2 - 0.7			3.4 - 8.8		
**pT1**				0.397			0.659			0.106
	93 (56)	43	50		0.2	0.6		2.8	8.0	
		16	106		0	2.6		0.4	49	
**pT2- 4**		44 - 56			0.3 - 0.8			5.2 - 11		

(*Continued*)

Parameters	n (%)	miR-101			miR-372			miR-373		
		relative values		p values	relative values		p values	relative values		p values
		Median	Mean		Median	Mean		Median	Mean	
	74 (44)	39	47		0.2	0.6		4.3	9.1	
		16	110		0	3		0.5	37	
**Grading**		41 - 54			0.3 - 0.9			5.7 - 12.4		
**1.2**				0.624			0.525			0.625
	98 (60)	42	47		0.3	0.6		3.6	8.7	
		15	100		0	2.9		0.3	49	
**3**		42 - 53			0.4 - 0.9			5.8 - 12		
	64 (40)	40	46		0.2	0.6		4.1	8.5	
		16	106		0	3.3		0.7	43	
**Estrogen receptor status**		40 - 53			0.3 - 0.8			5.2 - 12		
**neg.**				0.596			0.484			0.735
	38 (23)	42	50		0.2	0.5		4.1	7.4	
		18	123		0	3.3		0.7	31	
**pos.**		41 - 60			0.2- 0.9			3.6 - 11		
	127 (77)	41	48		0.2	0.6		3.4	8.9	
		14	102		0	2.9		0.3	48	
**Progesterone receptor status**		43 - 53			0.4 - 0.8			6.3 - 11		
**neg.**				0.672			0.479			0.095
	68 (41)	38	50		0.3	0.7		4.2	8.3	
		19	127		0	3.4		0.8	32	
**pos.**		42 - 59			0.4			5.4 - 11		
	98 (59)	44	48		0.2	0.5		2.7	8.6	
		14	100		0	1.9		0.4	49	
**HER2 status**		43 - 53			0.3 - 0.7			5.6 - 12		
**neg.**				0.829			0.644			0.548
	129 (82)	43	49		0.2	0.6		3.6	7.7	
		15	106		0	3.2		0.4	43	
**pos.**		43 - 55			0.4 - 0.8			5.5 - 10		
	28 (18)	40	40		0.2	0.4		3.8	5.4	
		16	117		0	2.4		0.3	15	
**Subtyp**		32 - 49			0.1 - 0.7			3.4 - 7.4		
**Luminal**				0.630			0.362			0.445
	119 (76)	41	47		0.2	0.6		3	7.9	

(Continued)

Parameters	n (%)	miR-101			miR-372			miR-373		
		relative values		p values	relative values		p values	relative values		p values
		Median	Mean		Median	Mean		Median	Mean	
		14	103		0	3.1		0.3	41	
HER2 pos.		41 - 53			0.4 - 0.8			5.5 - 10		
	5 (3)	42	33		0.1	0.7		4.2	6.7	
		15	-		0	-		1.2	-	
Triple neg.		6.2 - 60			0 - 2.7			0 - 19		
	33 (21)	43	53		0.2	0.5		4.4	8.4	
		21	142		0	3		0.8	40	
Overall survival		39 - 66			0.1 - 0.8			3.6 - 13		
alive				0.691			0.586			0.361
	147 (90)	43	49		0.2	0.6		3.6	8.8	
		16	104		0	3		0.4	48	
dead		44 - 54			0.4 - 0.8			6.4 - 11.1		
	16 (10)	40	46		0.3	0.3		3.8	4.1	
		5.7	-		0	-		0.6	-	
Disease-free survival		30 - 62			0.1 - 0.4			2.5 - 5.7		
Recurrence-free				*			0.318			*
	147 (90)	43	51		0.2	0.6		4.2	8.9	
		17	110		0	3		0.5	48	
Recurrence		46 - 56			0.4 - 0.8			6.5 - 11.3		
	16 (10)	35	39		0.2	0.3		2.9	3.3	
		10	-		0	-		0	-	
		29 - 49			0.1 - 0.6			1.7 - 4.9		
Healthy										
	28 (100)	20	24	**0.0001**	0.1	0.3	0.509	1.5	2.3	**0.001**
		7.9	51		0	-		0.1	6.5	
Benign		19 - 28			0 - 0.6			1.5 - 3.1		
	19 (100)	30	35	**0.013**	0.3	0.4	0.495	7.8	9.6	**0.024**
		16	-		0	-		0.3	-	
		25 - 45			0.1 - 0.7			6.1 - 13.0		

*no statistitical evaluation possible

significant p values in bold

Supplementary Table S2. Patient characteristics at the time of primary diagnosis of breast cancer and correlation to the relative miR concentrations in exosomes.

Parameters	n (%)	miR-101			miR-372			miR-373		
		relative values		p values	relative values		p values	relative values		p values
		Median	Mean		Median	Mean		Median	Mean	
		5%	95%		5%	95%		5%	95%	
		CI			CI			CI		
**Total**	50 (100)	101	118		1.1	1.6		16	29	
	38	271		0	4.7		1.5	104		
		99 - 137			1.2 - 2.0			20 - 38		
**Age**	56 (30-83)									
**Histology**				0.759			0.883			0.217
**ductal**	38 (76)	102	120		1.3	1.6		13	26	
		32	295		0	4.4		1.2	88	
		97 - 142			1.1 - 2.1			16 - 36		
**ductu-lobular**	2 (4)	81	81		1.0	1.0		33	33	
		64	-		0	-		13	-	
		0 - 286			0 - 13			0 - 283		
**lobular**	7 (11)	129	119		0.8	2.0		35	62	
		63	-		0.7	-		14	-	
		66 - 172			0 - 4.9			1.7 - 122		
**others**	3 (6)	73	71		0.9	0.9		13	56	
		69	-		0.8	-		7.0	-	
		44 - 98			0 - 2.3			0 - 677		
**Lymph node metastasis**				0.212			1.000			0.276
**neg.**	35 (71)	118	130		1.2	1.5		14	22	
		29	297		0	5.4		1.2	112	
		100 - 160			0.9 - 2.1			11 - 32		
**pos.**	14 (29)	90	90		1.2	1.3		29	37	
		63	-		0	-		1.6	-	
		79 - 101			0.8 - 1.9			16 - 59		
**Tumor stage**				0.435			0.174			0.271
**pT1**	25 (50)	112	113		1.0	1.2		13	20	
		21	289		0	4.3		1.4	92	
		95 - 132			0.6 - 1.9			10 - 30		
**pT2- 4**	25 (50)	83	119		1.6	1.6		17	35	
		40	280		0	-		1.1	132	

(*Continued*)

Parameters	n (%)	miR-101			miR-372			miR-373		
		relative values		p values	relative values		p values	relative values		p values
		Median	Mean		Median	Mean		Median	Mean	
		78 - 160			1.1 - 2.2			17 - 53		
**Grading**				0.377			0.636			0.133
**1.2**	27 (61)	97	112		1.2	1.5		14	22	
		21	240		0	5.8		1.0	122	
		85 - 138			0.8 - 2.2			10 - 34		
**3**	17 (39)	101	121		1.0	1.3		21	33	
		63	-		0	-		7.0	-	
		86 - 156			0.7 - 1.9			16 - 49		
**Estrogen receptor status**				0.242			0.852			**0.021**
**neg.**	14 (29)	108	127		1.3	1.4		24	45	
		43	-		0	-		9.7	-	
		72 - 181			0.5 - 2.2			20 - 69		
**pos.**	35 (71)	100	111		1.1	1.5		13	19	
		29	240		0	5.3		1.2	112	
		90 - 133			0.9 - 2.0			11 - 28		
**Progesterone receptor status**				0.488			0.851			**0.013**
**neg.**	22 (44)	97	113		1.2	1.3		23	39	
		35	304		0	-		6.2	103	
		75 - 151			0.7 - 2.0			20 - 57		
**pos.**	28 (56)	112	118		1.1	1.5		13	18	
		28	243		0	5.6		1.0	124	
		93 - 144			0.9 - 2.1			8.5 - 27		
**HER2 status**				0.668			0.608			0.492
**neg.**	36 (80)	113	121		1.0	1.5		16	27	
		50	257		0	5.1		1.2	112	
		97 - 144			1.0 - 2.0			16 - 38		
**pos.**	9 (20)	100	87		1.3	1.1		23	28	
		45	-		0.8	-		5.6	-	
		66 - 108			0.7 - 1.5			0 - 69		
**Subtyp**				0.424			0.941			0.074
**Luminal**	29 (66)	100	111		1.1	1.5		13	19.3	
		29	240		0	5.3		1.2	112	
		90 - 133			0.9 - 2.0			11 - 28		

(*Continued*)

Parameters	n (%)	miR-101			miR-372			miR-373		
		relative values		p values	relative values		p values	relative values		p values
		Median	Mean		Median	Mean		Median	Mean	
**HER2 pos.**	3 (7)	120	-		1.3	-		23	-	
		79	-		1.3	-		10	-	
		-		-		-				
**Triple neg.**	12 (27)	108	132		1.0	1.4		23	43	
		43	-		0	-		10	-	
		71 - 194			0.3 - 2.4			15 - 70		
**Overall survival**				*			*			*
**alive**	45 (94)	100	116		1.2	1.5		17	28	
		36	282		0	4.9		1.4	104	
		95 - 137			1.0 - 1.9			18 - 38		
**dead**	3 (6)	118	-		0.9	-		16	-	
		63	-		0	-		13	-	
			-			-			-	
**Disease free survival**				*			*			*
**Recurrence free**	45 (96)	101	117		1.1	1.5		16	27	
		36	282		0	4.2		1.4	96	
		95 - 139			1 - 1.9			16 - 37		
**Recurrence**	2 (4)	99	99		0.6	0.6		37	37	
		79	-		0	-		13	-	
		0 - 347			0 - 8.8			0 - 342		
**Healthy**	12 (100)	55	56	**0.0001**	0.4	0.6	**0.021**	12	11	0.082
		27	-		0	-		1.0	-	
		43 - 68			0 - 1.1			6.6 - 15		

*no statistitical evaluation possible

significant p values in bold
